# Phytochemical profile and antiproliferative effect of *Ficus crocata* extracts on triple-negative breast cancer cells

**DOI:** 10.1186/s12906-020-02993-6

**Published:** 2020-06-22

**Authors:** Carlos A. Sánchez-Valdeolívar, Patricia Alvarez-Fitz, Ana E. Zacapala-Gómez, Macdiel Acevedo-Quiroz, Lorena Cayetano-Salazar, Monserrat Olea-Flores, Jhonathan U. Castillo-Reyes, Napoleón Navarro-Tito, Carlos Ortuño-Pineda, Marco A. Leyva-Vázquez, Julio Ortíz-Ortíz, Yaneth Castro-Coronel, Miguel A. Mendoza-Catalán

**Affiliations:** 1 Facultad de Ciencias Químico Biológicas, Universidad Autónoma de Guerrero, Av. Lázaro Cárdenas, Ciudad Universitaria, 39090 Chilpancingo, Guerrero Mexico; 2 CONACYT-Universidad Autónoma de Guerrero, Chilpancingo, Guerrero Mexico; 3 Tecnológico Nacional de México, Instituto Tecnológico de Zacatepec, Calzada Tecnológico 27, Centro, 62780 Zacatepec, Morelos Mexico

**Keywords:** *Ficus crocata*, Moraceae, Breast cancer, Apoptosis, MDA-MB-231 cells

## Abstract

**Background:**

Some species of the *Ficus* genus show pharmacological activity, including antiproliferative activity, in cell lines of several cancer Type*s. ficus crocata* is distributed in Mexico and used in traditional medicine, as it is believed to possess anti-inflammatory, analgesic, and antioxidant properties. However, as of yet, there are no scientific reports on its biological activity. This study aims to evaluate the phytochemical profile of *F. crocata* leaf extracts and their effects on breast cancer MDA-MB-231 cells proliferation. Moreover, the study aims to unearth possible mechanisms involved in the decrease of cell proliferation.

**Methods:**

The extracts were obtained by the maceration of leaves with the solvents hexane, dichloromethane, and acetone. The phytochemical profile of the extracts was determined using gas chromatography coupled with mass analysis. Cell proliferation, apoptosis, and cell cycle analysis in MDA-MB-231 cells were determined using a Crystal violet assay, MTT assay, and Annexin-V/PI assay using flow cytometry. The data were analyzed using ANOVA and Dunnett’s test.

**Results:**

The hexane (Hex-EFc), dichloromethane (Dic-EFc), and acetone (Ace-EFc) extracts of *F. crocata* decreased the proliferation of MDA-MB-231 cells, with Dic-EFc having the strongest effect. Dic-EFc was fractioned and its antiproliferative activity was potentiated, which enhanced its ability to induce apoptosis in MDA-MB-231 cells, as well as increased p53, procaspase-8, and procaspase-3 expression.

**Conclusions:**

This study provides information on the biological activity of *F. crocata* extracts and suggests their potential use against triple-negative breast cancer.

## Background

Breast cancer is the most common cancer and the main cause of death for women worldwide. It represents about 12% of all new cancer cases and 25% of all cancers in women [1]. Triple-negative breast cancer (TNBC) represents 10–20% of all breast carcinomas [2] and is characterized by no expression of estrogen receptors (ER) and progesterone receptors (PR), and the lack of overexpression of the HER2 protein. Thus, the tumoral cells do not respond to hormone therapy, and it is considered the subtype with the worst prognosis among breast cancer cases [3–5]. Among the different drugs used in cancer, many of these are derived from plants and some are utilized in their natural form or with structural modifications, such as polyphenols (e.g., resveratrol [6], epicathechin [7], and epigallocatechin gallate [8]) and flavonoids (e.g., quercetin [9], silibinin [10], and oncamex [11]), which, in cancer cells, have demonstrated the ability to induce apoptosis [12–15].

*Ficus* genus species are employed in traditional medicine for the treatment of asthma, migraine, cough, diarrhea, earache, toothache, scabies, and eye problems [16–21]. Several studies have reported that some species of *Ficus* possess pharmacological activities, such as antioxidant [20, 22–26], antimicrobial [26–29], antiviral [30–32], anti-inflammatory [33–36], antiparasitic [20, 37], antidiabetic [25, 38–42], antiproliferative [28, 43–53], and cytotoxic activities [32, 53–57]. Extracts of *Ficus religiosa* have exhibited cytotoxic properties, inducing apoptosis in cervical cancer HeLa cell lines and cell cycle arrest in SiHa cells [45]. Moreover, *Ficus fistulosa*, *Ficus hispida*, and *Ficus schwarzii* have shown antiproliferative activity in human brain glioblastoma (U87MG), lung adenocarcinoma (A549), and colorectal adenocarcinoma (HT-29) cell lines [43]. The biological properties of *Ficus* species are attributed to the wide range of secondary metabolites identified in the root, stem, leaf, bark, and fruit, which are mostly alkaloids, flavonoids, coumarins, phenols, steroids, terpenoids, and triterpenoids [9, 16, 18, 21, 23–26, 39, 40, 51, 52, 54, 56, 58, 59]. In Mexico, the presence of 21–40 species of *Ficus* has been reported, among which 13 species have been identified in southern Mexico, including *Ficus crocata* [60–62]. However, there are no reports on the biological activity of these species of *Ficus*. This study aimed to evaluate the phytochemical profile of leaf extracts of *F. crocata* and the effect of the exposure of breast cancer cells MDA-MB-231 to these extracts. Moreover, cell proliferation and the possible mechanisms involved in the decrease of proliferation, such as apoptosis and cell cycle arrest, were investigated.

## Methods

### Plant material

Leaves of *F. crocata* were collected from the wild in Petaquillas, Guerrero State, Mexico (latitude: 17.3708, longitude: − 99.5344, altitude: 1160 masl); in accord with Mexican official standard NOM-059-SEMARNAT-2010, there are no restrictions for the collection of this species. The plant was authenticated by Blanca Verónica Juárez-Jaime, M.Sc., and Mauricio Mora-Jarvio, B. Sc, biologists from Herbario Nacional de México (MEXU). A voucher specimen (MEXU-2052) was deposited at the same institute.

### Preparation of extracts and fractionation

Leaves of *F. crocata* (100 g) were dried and ground, and then successively macerated (sequential extraction) with hexane, dichloromethane, and acetone solvents (reactive-grade, 500 mL during 24 h, three times). The macerated material was filtered, and the organic phase was evaporated in a rotary evaporator (Digital Rotary Evaporator Model 410) at 60 °C and 80 rpm. Hexane (1.51% yield; Hex-EFc), and dichloromethane (0.94% yield; Dic-EFc), and acetone (11.75% yield; Ace-EFc) extracts were stored at − 20 °C and protected from light.

Dic-EFc was subjected to fractionation by Open glass Column Chromatography (OCC, ID: 20 mm) packed with 30 g of silica gel (7734, 60 Å, 70–230 mesh; Sigma-Aldrich). The procedure was initiated with the hexane–acetone 9:1 elution system and the polarities were gradually increased with acetone until finally obtaining 149 aliquots (10 mL). Aliquots were analyzed by TLC (silica gel 60, F_254_, 20 × 20 cm; Merck, Germany) and visualized by UV light at 254 and 365 nm and were combined according to the characteristics observed by TLC in 17 fractions (A1/A17-Dic-EFc). The fractions were monitored by thin layer chromatography (TLC) (Silica gel 60 F254; Merck, Germany) and visualized by ultraviolet (UV) light at 254 nm, then revealed with vanillin-sulfuric acid, vanillin-phosphoric acid, Dragendorff reagent, Liebermann–Burchard reagent, potassium hydroxide reagent (KOH), and acid developer [63].

### Gas chromatography coupled with mass analysis (GC-MS)

GC-MS analyses were carried in triplicate out on an Agilent 6890 series gas chromatograph equipped with a mass selective detector 5973 N (USA). The experimental conditions of GC-MS system were as follows [64]: HP-5MS capillary nonpolar column (30 m, ID: 0.20 mm, film thickness: 0.25 μm). The carrier gas was helium at flow rate of 1.0 mL/min. In the gas chromatography part, the temperature program (oven temperature) was 50 °C raised to 230 °C at 2 °C/min and the injection volume was 1 μL. Samples were dissolved in dichloromethane. All results were compared by using NIST/EPA/NIH Mass Spectral library version 1.7a/ChemStation.

### Cell culture and exposure to extracts

MDA-MB-231 cells (ATCC® HTB-26) were cultured in Dulbecco’s Modified Eagle Medium Formula 12 (DMEM/F12) supplemented with 5% Fetal Bovine Serum (FBS), 1% antibiotic (ampicillin/streptomycin), and incubated at 37 °C in a 5% CO_2_ atmosphere and at 95% humidity. Cells were synchronized with basal medium without FBS for 24 h and exposed to 0–80 μg mL^− 1^ of *F. crocata* extracts for 24–48 h. DiMethyl SulfOxide (DMSO) was used as the diluent of extracts (Vehicle). As a positive control, cells were treated with 100 μM Cytarabine (Ara-C), which is known to induce cell death [65]. As a negative control, cells were treated with 5% FBS to induce cell proliferation. All tests were performed in triplicate at three independent times.

### Cell growth in monolayer (crystal violet assay)

A total of 15 × 10^3^ cells were plated on 24-well plates (Corning) and cultured in DMEM/F12 medium supplemented with 5% FBS for 24 h. Cells were treated with 0, 5, 10, 20, 40, and 80 μg mL^− 1^ of *F. crocata* extracts for 24 and 48 h, fixed after treatment in 4% formaldehyde for 5 min, and the cell morphology was observed using an inverted microscope (EVOS Cell Imaging System; Thermo Scientific). Later, the cells were stained with 0.5% Crystal violet, the excess dye was washed with water and PBS (Phosphate-Buffered Saline) and the stain was extracted with 10% acetic acid. The relative cell density in the monolayer was determined by measuring the optical density (OD) of each well at 600 nm in a biophotometer (Eppendorf Model RS-2312 DH 8.5 mm).

### MTT assay

The percentage of viable cells was evaluated using the MTT cell proliferation colorimetric assay (CT02, Millipore Corp., Bedford, MA, USA) according to the manufacturer’s instructions. Briefly, in a 96-well plate, 1 × 10^4^ MDA-MB-231 cells per well were cultured for 24 h with DMEM/F12 medium with 5% FBS and subsequently with basal medium for 24 h to synchronize the cells in the G1 phase and promote a homogeneous cellular response. Then, the treatment with 0–80 μg mL^− 1^ of *F. crocata* extracts or fractions was applied for 24 and 48 h. After the treatment, the medium containing the extracts was replaced by fresh basal medium and 100 μL of the MTT reagent was added for 4 h. The formazan crystals were diluted with isopropanol, and the OD of the supernatant was obtained at a wavelength of 540 nm using a biophotometer (Eppendorf Model RS-2312 DH 8.5 mm). The half-maximal inhibitory concentration (IC_50_) was calculated through the linear eq. (Y = mX + b) using GraphPad prism software v6.0.

### Analysis of apoptosis

In order to determine the cell population in apoptosis after the treatment with *F. crocata* extracts, the Fluorescein Isothiocyanate (FITC) Annexin V Apoptosis Detection Kit I (556,547; Beckton Dickinson) was used according to the manufacturer’s instructions. Briefly, MDA-MB-231 cells were seeded at a density of 2 × 10^5^ cells/well in a six-well plate for 24 h with DMEM and 5% FBS. The cells were synchronized with basal medium for 24 h and subsequently exposed to Dic-EFc (0, 20, 40, and 80 μg mL^− 1^) or A9, A12, and A13-Dic-EFc fractions (80 μg mL^− 1^) for 48 h. Cells were collected by trypsinization followed by washing with PBS, and staining with FITC Annexin V and propidium iodide (PI) for 15 min in the dark. They were then immediately analyzed by flow cytometry (FACSCanto II; Beckton Dickinson, USA). The FITC Annexin V- and PI-negative cells were considered viable cells. Cells in early apoptosis were FITC Annexin V-positive and PI-negative, while cells in late apoptosis were both FITC Annexin V- and PI- positive.

### Analysis of cell cycle arrest

MDA-MB-231 cells were seeded at a density of 2 × 10^5^ cells/well in a six-well plate and incubated for 24 h in an incubator at 37 °C with a 5% CO_2_ atmosphere. The cells were synchronized in basal medium for 24 h and subsequently exposed to Dic-EFc (0, 20, 40, and 80 μg mL^− 1^) or A9, A12, and A13-Dic-EFc fractions (80 μg mL^− 1^) for 48 h. Cells were collected by trypsinization followed by washing with PBS, centrifugation, and fixing with ethanol. Subsequently, the cells were centrifuged and resuspended in cold PBS. A total of 20 μg mL^− 1^ RNAsa was added for 30 min and, the cells were stained with Propidium Iodide (PI) in the dark at room temperature for 15 min. The immunofluorescence of PI was analyzed by flow cytometry (FACSCanto II; Beckton Dickinson).

### Immunobloting for p53 and caspases

After cell stimulation with the Dic-EFc or A9- Dic-EFc fraction, the cells were solubilized in 0.2 mL of RIPA buffer (50 mM HEPES pH 7.4, 150 mM NaCl, 1 mM EDTA/EGTA, 10 mM sodium pyrophosphate, 10% glycerol, 1% Triton X-100, 1% sodium deoxycholate, 1.5 mM MgCl_2_, 0.1% SDS, 100 mM NaF, 1 mM sodium orthovanadate and 1 mM PMSF). Whole cell lysates (20 μg) were resolved on 10% SDS-polyacrylamide gels and transferred to nitrocellulose membranes. The membranes were incubated in 5% non-fat dried milk in PBS for 1 h at room temperature (RT), and incubated with the primary antibodies anti-p53 (Sc-126, SC Biotech, 1:2000 dilution), anti-caspase-3 (which also recognizes other caspases in according to cell type) (H-277, sc-7148, 1:2000 dilution), and anti-GAPDH (AB clonal AC001, 1:5000 dilution) at 4 °C overnight. Later, the membranes were incubated for 2 h at RT with secondary anti-mouse or anti-rabbit (1:5000 dilution, MERCK Millipore), and revealed with a chemiluminescent substrate from Bio-Rad (Hercules, CA, USA).

### Statistical analysis

Data analysis was performed using the GraphPad Prism version 7.0 statistical software. The data were shown as the mean ± standard deviation (SD). One-way analysis of variance (ANOVA) was used with Dunnett’s test. A statistically significant difference was considered when *p* < 0.05.

## Results

### Phytochemical analysis

The GC-MS analysis of Hex-EFc, Dic-EFc and Ace-EFc extracts revelated the existence of 19, 10 and 6 phytochemical compounds, respectively (Table 1). The most prevailing compounds were the terpenes lup-20(29)-en-3-ol acetate (28.39%) and lupeol (18.61%) in Hex-EFc; lupeol (27.86%), the fatty acid 10,13,13-trimethyl-11-tetradecen-1-ol acetate (14.80%), and lup-20 (29)-en-3-ol acetate (10.90%) in Dic-EFc; and β-sitosterol (42.36%) and stigmastan-3,5-dien (23.87%) in Ace-EFc. Compounds identification was established based on the peak area (%), and retention time (RT) (Table 1). The chemical structures of the molecules identified are expressed in Figs. S1, S2, and S3.

**Table 1:** The chemical composition of extracts from *F. crocata* using GC-MS.

Plant extract	Name of compound	Molecular formula	Molecular weight	Peak area
**Hex-EFc**			(g/mol)	(%)
1	3,7,11,15-tetramethyl-2,1-hexadecen 1-ol	C_20_H_40_O	296.539	0.120
2	2-pentadecanone,6,10,14-trimethyl-	C_18_H_36_O	268.485	0.404
3	1-dodecanol 3,7,11-trimethyl	C_15_H_32_O	228.42	0.174
4	hexadecanoic acid, ethyl ester	C_18_H_36_O_2_	284.484	0.096
5	4,8,12,16-tetramethyl-heptadecan-4-olide	C_21_H_40_O_2_	324.549	0.314
6	2H-pyran-ona, tetrahydro-6-trydecyl	C_18_H_34_O_2_	282.468	1.470
7	octacosane	C_28_H_58_	394.772	8.697
8	hexatriacontane	C_36_H_74_	506.988	9.133
9	campesterol	C_28_H_48_O	400.691	0.625
10	stigmasterol	C_29_H_48_O	412.702	1.334
11	β-sitosterol	C_29_H_50_O	414.718	5.288
12	13,27-cycloursane	C_30_H_50_O	410.718	1.874
13	α-amyrin	C_30_H_50_O	426.729	1.308
14	lup-20 (29)-en-3-one	C_30_H_48_O	424.713	4.665
15	lupeol	C_30_H_50_O	426.729	18.610
16	13,27–3-cycloursan-3-ol acetate, (3β,13β,14β)	C_32_H_52_O_2_	468.754	4.608
17	lup-20 (29)-en-3-ol, acetate (3β)	C_32_H_52_O_2_	468.7541	28.386
18	friedelin	C_30_H_50_O	426.729	4.335
19	9,19-cyclolanostan-3-ol, 24, methylene, acetato, (3β)	C_31_H_52_O	440.756	1.691
**Dic-EFc**				
1	acetic acid, 2 (2,2,6 trimethyl-7-oxa-bicyclo-(4,10)-hept-1-yl propenyl ester	C_14_H_22_O_3_	238.323	5.748
2	2-pentadecanone,6,10,14-trimethyl-	C_18_H_36_O	268.485	6.994
3	3,7,11,15-tetramethyl-2,1-hexadecen 1-ol	C_20_H_40_O	296.539	4.174
4	10,13,13-trimethyl-11-tetradecen-1-ol acetate	C_19_H_36_O_2_	296.495	14.808
5	octadecanal 2-bromo	C_18_H_35_BrO	347.381	0.915
6	4,25-secoobscurinervan, 21-deoxy-16methoxy-22-methyl-(22 α)	C_23_H_22_N_2_O_2_	368.512	0.911
7	stigmasterol	C_29_H_48_O	412.702	1.247
8	β-sitosterol	C_29_H_50_O	414.718	5.121
9	lupeol	C_30_H_50_O	426.729	27.861
10	lup-20 (29)-en-3-ol, acetate (3β)	C_32_H_52_O_2_	468.7541	10.903
**Ace-EFc**				
1	3,7,11,15-tetramethyl-2,1-hexadecen 1-ol	C_20_H_40_O	296.539	1.700
2	squalene	C_30_H_50_	410.73	0.547
3	stigmastan-3,5-dien	C_29_H_48_	396.703	23.877
4	alpha-tocopherol	C_29_H_50_O_2_	430.717	8.627
5	β-sitosterol	C_29_H_50_O	414.718	42.363
6	lupeol	C_30_H_50_O	426.729	6.961

### Leaf extracts of *F. crocata* decreased the proliferation of MDA-MB-231 cells

**Fig. 1:**
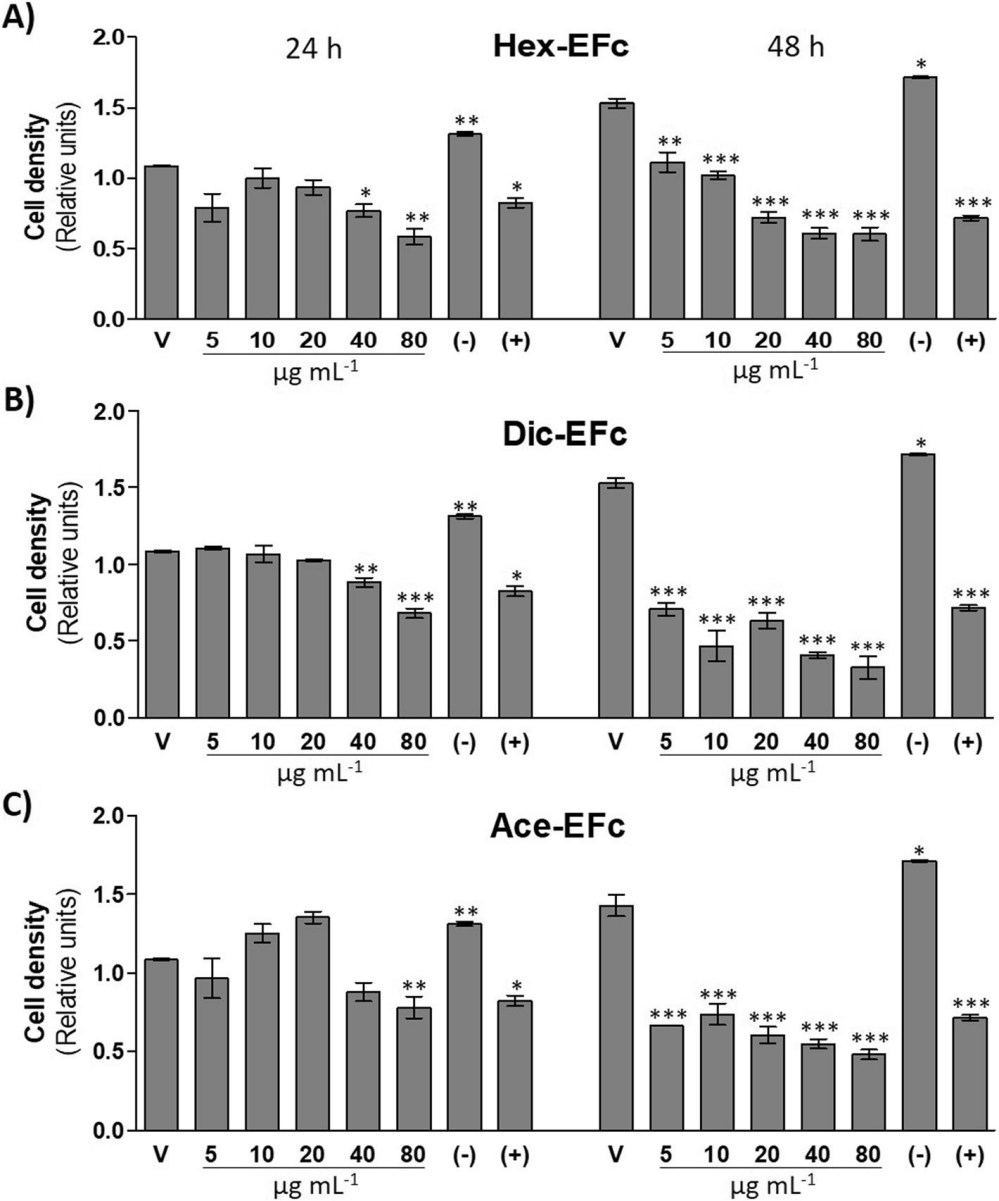
Effect of extracts of *Ficus crocata* on the growth in a monolayer of MDA-MB-231 cells. Crystal violet assay; **a** Hex-EFc: hexane extract of *F. crocata*. **b** Dic-EFc: dichloromethane extract of *F. crocata*. **c** Ace-EFc: acetone extract of *F. crocata*. V: vehicle, DMSO. (−): negative control, 5% FBS. (+): positive control, 100 μM Ara-C (cytarabine); One-way ANOVA, Dunnett’s test: *p < 0.05, **p < 0.01 and ***p < 0.001 versus V

**Fig. 2:**
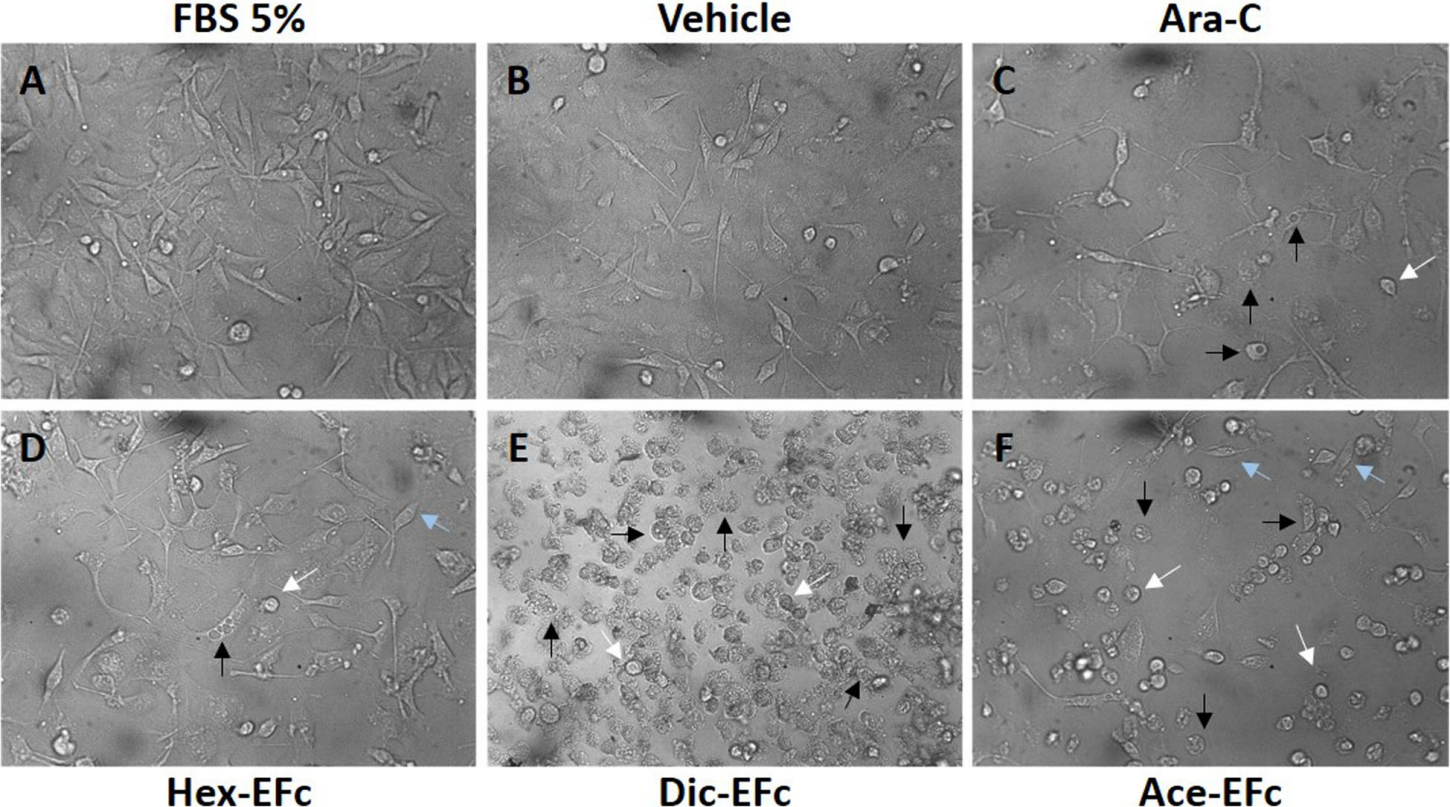
Morphologic changes in MDA-MB-231 cells exposed to *F. crocata* extracts. The images are representative of cells treated for 48 h with FBS 5% (**a**), V: Vehicle, DMSO (**b**), 100 μM Ara-C (cytosine β-D-arabinofuranoside) (**c**), and 20 μg mL^− 1^ of Hex-EFc (**d**), Dic-EFc (**e**), and Ace-EFc (**f**). The treatment induced a decrease in cell size (blue arrow), rounded shape (white arrow), and the formation of intracellular vacuoles (black arrow). 20× magnification

To evaluate the antiproliferative activity of *F. crocata* extracts on MDA-MB-231 breast cancer cells, crystal violet and MTT assays were performed. It was observed that Hex-EFc, Dic-EFc, and Ace-EFc decreased the number of cells in the monolayer in a concentration- and time-dependent manner. At 24 h of treatment, only 40 and 80 μg mL^− 1^ concentrations decreased the cell density compared to the untreated control cells (*p* < 0.001). The effect of extracts was greater at 48 h, showing a lower cell density in the monolayer at all of the tested concentrations and, decreasing the number of cells as the concentration increased (*p* < 0.001) (Fig. 1). In addition, Dic-EFc and Ace-EFc induced morphologic changes in MDA-MB-231 cells, such as a decrease in cell size, a rounded shape, and the formation of intracellular vacuoles suggestive of apoptosis (Fig. 2). These cellular changes were evident at 48 h with 5-, 10-, and 20-μg mL^− 1^ concentrations, mainly with Dic-EFc. Figure 2 shows the effect of the extracts at 20 μg mL^− 1^ after 48 h of treatment.

**Fig. 3:**
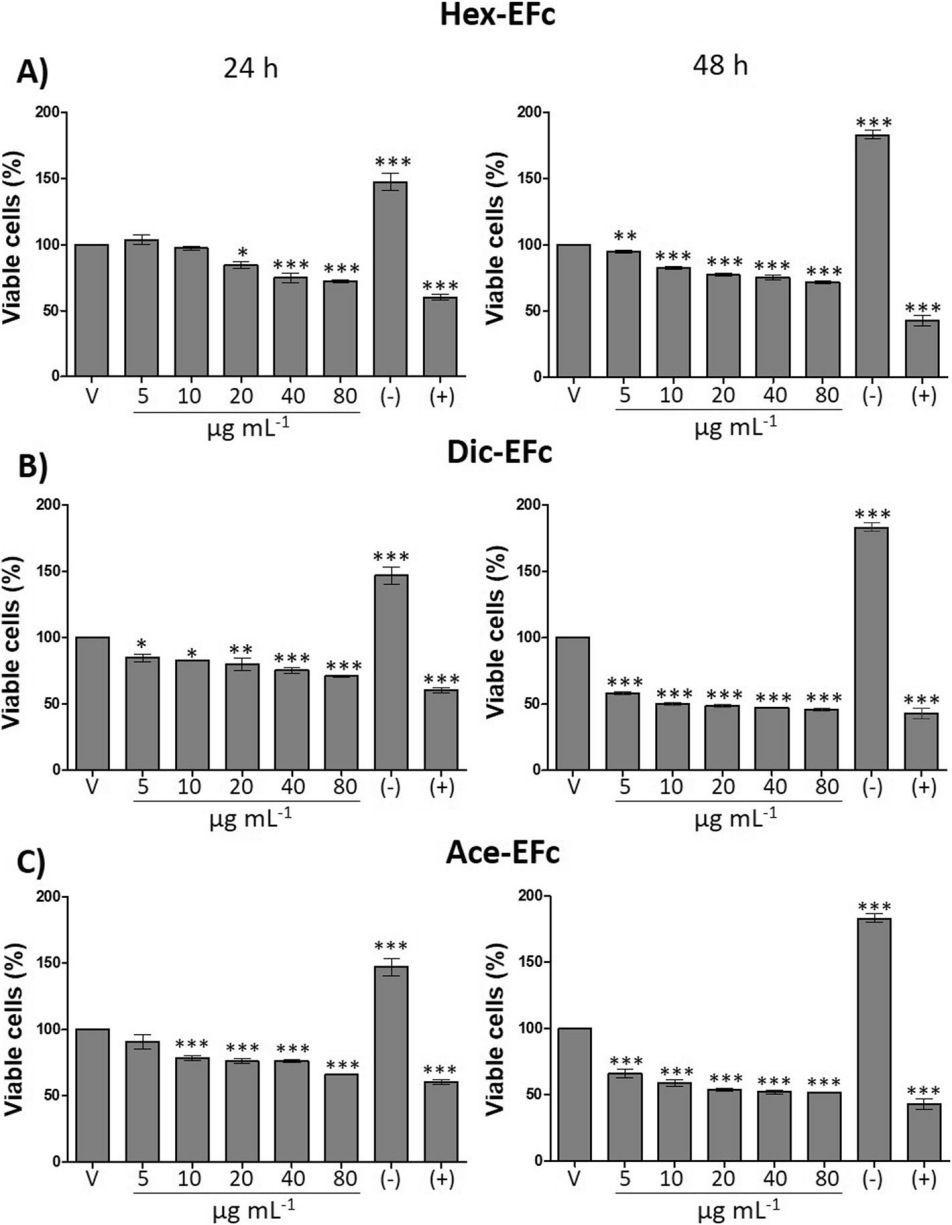
Effect of leaf extracts of *F. crocata* on MDA-MB-231 cell viability. MTT assay; V: vehicle, DMSO. (−): negative control, 5% FBS. (+): positive control, 100 μM Ara-C (cytarabine); **a** Hex-EFc: hexane extract of *F. crocata*. **b** Dic-EFc: dichloromethane extract of *F. crocata.***c** Ace-EFc: acetone extract of *F. crocata*. One-way ANOVA, Dunnett’s test: **p < 0.05, **p < 0.01 and ***p < 0.001 versus V

In order to validate the data observed with the Crystal violet assay, the effect of the extracts on cell proliferation was determined by MTT assay in order to consider only metabolically viable cells. After 24 h of treatment, a significant decrease in cell proliferation was observed with ≥5 μg mL^− 1^ of Dic-EFc, ≥10 μg mL^− 1^ of Ace-EFc, and ≥ 20 μg mL^− 1^ of Hex-EFc compared to untreated control cells. After prolonging exposure to extracts for 48 h, Hex-EFc, Dic-EFc, and Ace-EFc decreased cell proliferation at all tested concentrations compared to control cells (*p* < 0.001). However, this effect was more evident with Dic-EFc, which decreased the percentage of viable cells by more than 50% with 10–80 μg mL^− 1^ (*p* < 0.001) (Fig. 3). Antiproliferative activity at 48 h was greater for Dic-EFc (IC_50_: 32.43 μg mL^− 1^), followed by Ace-EFc (IC_50_: 78.49 μg mL^− 1^), and Hex-EFc (IC_50_: 164.05 μg mL^− 1^) (Fig. S4). In addition, the effect of DIC-EFc on viability of MCF-10A non-tumor cells was preliminary evaluated, and only 320 μg mL^− 1^ of extract showed cytotoxic effect at 48 h of treatment (Fig. S5).

**Fig. 4:**
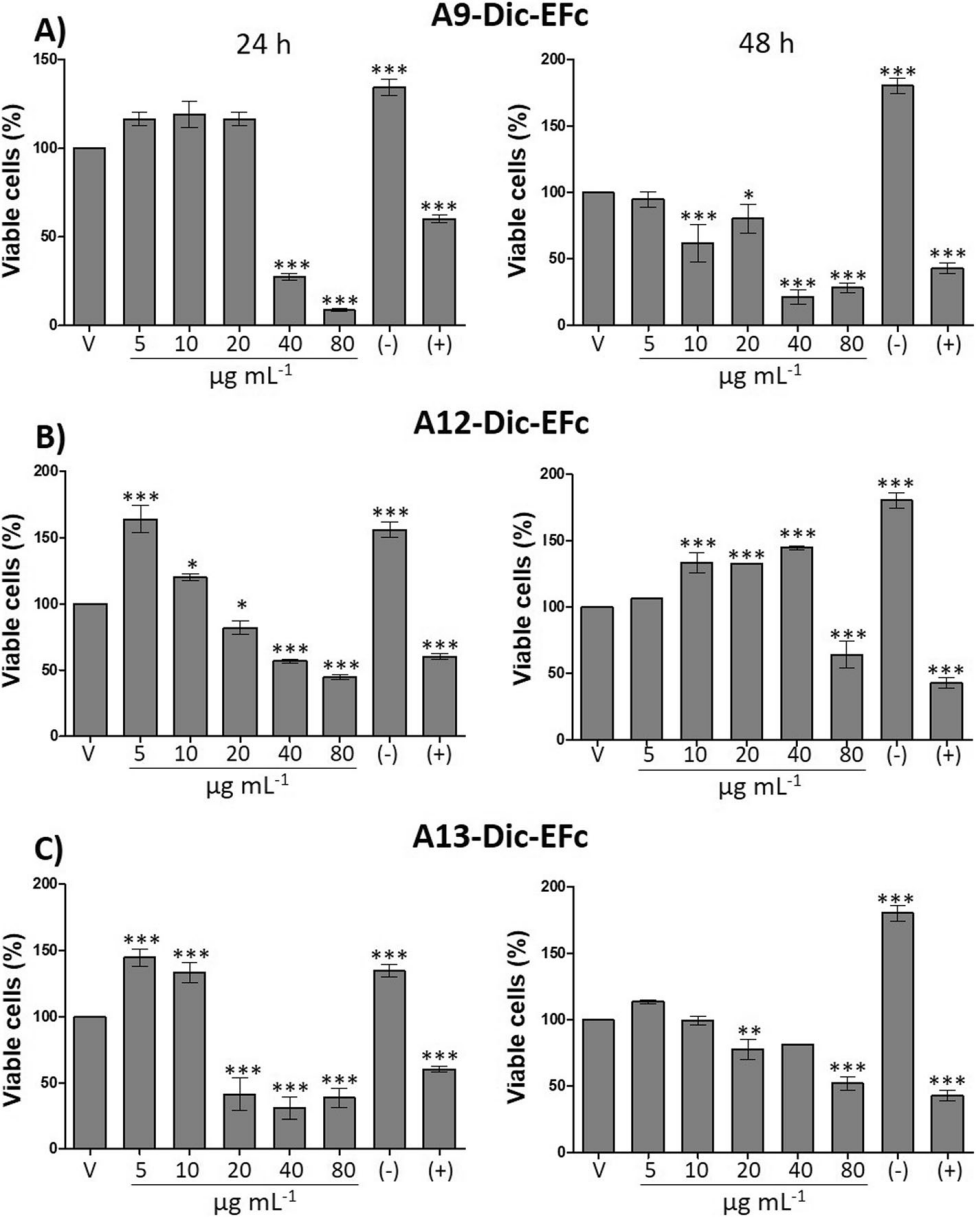
Effect of A9-, A12-, and A13-Dic-EFc fractions on MDA-MB-231 cells viability. MTT assay. **a** A9-Dic-EFc fraction, (**b**) A12-Dic-EFc fraction, (**c**) A13-Dic-EFc fraction. V: vehicle, DMSO. V: vehicle, DMSO. (−): negative control, 5% FBS. (+): positive control, 100 μM Ara-C (cytarabine); One-way ANOVA, Dunnett’s test: *p < 0.05, **p < 0.01 and ***p < 0.001 versus V

Considering that Dic-EFc exhibited the best effect in decreasing cell proliferation (IC_50_: 32.43 μg mL^− 1^), 17 fractions of Dic-EFc were obtained. The fractions demonstrated the presence of cardiotonic glycosides and anthraquinones (Table S1). Considering the phytochemical profile, three fractions (A9-Dic-EFc, A12-Dic-EFc, and A13-Dic-EFc) were selected to evaluate their effect on the proliferation of MDA-MB-231 cells. The A9-Dic-EFc fraction exhibited the presence of anthraquinones, triterpenoids, lignans, essential oils, phenylpropanoids, cucurbitacins, steroids, and saponins. The A12-Dic-EFc fraction revealed only the presence of anthraquinones, while the A13-Dic-EFc fraction contained lignans, anthraquinones, and cardiotonic glucosides (Table S1). Exposure to ≥40 μg mL^− 1^ of A9-Dic-EFc for 24 h decreased the number of viable cells by around 70% (*p* < 0.001) and this effect was maintained at 48 h of exposure. On the other hand, after 24 h of exposure to > 40 μg mL^− 1^ of A12-Dic-EFc, the cell proliferation was decreased by around 50%, and > 20 μg mL^− 1^ of A13-Dic-EFc decreased cell proliferation by around 60%. However, at 48 h, both fractions maintained their antiproliferative activity at a concentration of only 80 μg mL^− 1^ (Fig. 4). Low concentrations of the A9-Dic-EFc fraction (5–20 μg mL^− 1^) exhibited no effect on the proliferation of MDA-MB-231 cells, while low concentrations of A-12- and A13-Dic-EFc fractions (5–10 μg mL^− 1^) appeared to stimulate cell proliferation after 24 h (A12- and A13-Dec-EFc) or 48 h of exposure (A12-Dec-EFc) (Fig. 4 b and c). Considering the above, the A9-Dic-EFc fraction showed a better effect for decreasing cell proliferation and this observation could be explained by differences in the phytochemical profile of A9-Dic-EFc with respect to the A12- and A13-Dic-EFc fractions described previously (Table S1). Antiproliferative activity against MDA-MB-231 cells after 48 h of exposure was greatest for the A9-Dic-EFc fraction (IC_50_: 39.89 μg mL^− 1^), followed by the A13-Dic-EFc fraction (IC_50_: 80.49 μg mL^− 1^) and the A12-Dic-EFc fraction (IC_50_: 126.76 μg mL^− 1^) (Fig. S4).

### The dichloromethane extract of *F. crocata* induced apoptosis and cell cycle arrest in MDA-MB-231 cells

**Fig. 5:**
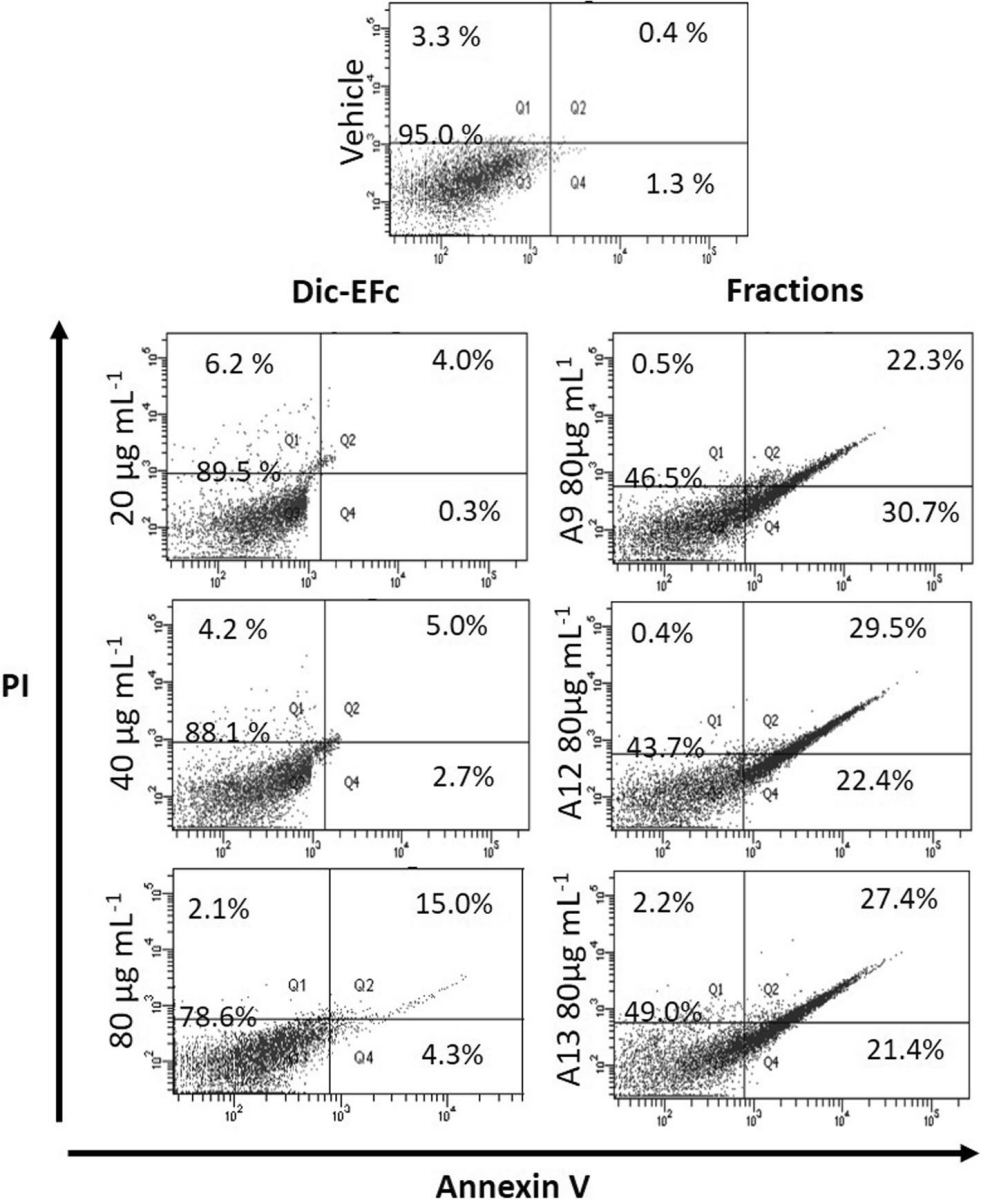
Dic-EFc and the A9-, A12-, and A13-Dic-EFc fractions induced apoptosis in MDA-MB-231 cells. Representative pictograms of cells treated as indicated for 48 h. Q3: viable cells. Q4: Annexin V-positive (early-apoptotic cells). Q2: Annexin V/PI double-positive (late-apoptotic cells). Q1: dead cells (no apoptosis). Vehicle: cells treated with 1% DMSO

**Fig. 6:**
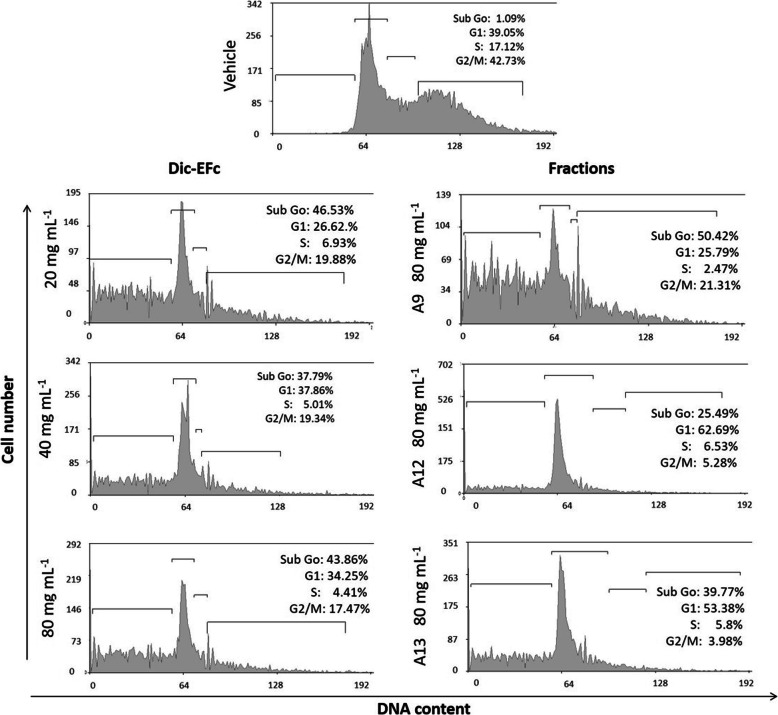
Effect of treatment with Dic-EFc and the A9-, A12-, and A13-Dic-EFc fractions on the cell cycle of MDA-MB-231 cells. Representative pictograms of cells treated as indicated for 48 h. Vehicle: cells treated with 1% DMSO

To determine possible mechanisms to reduce the proliferation of MDA-MB-231 cells exposed to the dichloromethane extract of *F. crocata*, an apoptosis assay and cell cycle analysis were performed. Only the concentrations of Dic-EFc (20–80 μg mL^− 1^) and its fractions (80 μg mL^− 1^) that reduced around 50% or more of cell proliferation were analyzed. It was observed that the Dic-EFc treatment induced apoptosis in 4.3 and 7.7% of the cell population with 20 μg mL^− 1^ and 40 μg mL^− 1^, respectively, while 80 μg mL^− 1^ increased the apoptotic population to 19.3% (4.3 and 15% in early and late apoptosis, respectively). Interestingly, the fractionation of Dic-EFc components enhanced the ability of the extract to induce apoptosis in MDA-MB-231 cells. It was observed that A9-Dic-EFc, A12-Dic-EFc, and A13-Dic-EFc at 80 μg mL^− 1^ all induced apoptosis. The A9-Dic-EFc fraction showed the highest percentage of apoptotic cells with 53% (30.7% in early apoptosis and 22.3% in late apoptosis), followed by the A12-Dic-EFc and A13-Dic-EFc fractions, with 51.9 and 48.8%, respectively (Fig. 5). Moreover, a decrease was observed in the cell population in the S and G2/M phases of the cell cycle, and an increase during the sub-G0 phase was noted after treatment with Dic-EFc and its fractions A9, A12, and A13. A highlighted effect was observed with fraction A9, with more than 50% of the cell population occurring during the sub-G0 phase (Fig. 6). This high percentage in the sub-G0 phase was consistent with the apoptosis results observed with the Annexin/PI assay.

Exposure to A12-Dic-EFc and A13-Dic-EFc fractions caused an accumulation of G1 phase cells of 62 and 53%, respectively, suggesting that metabolites in these fractions induce a G1-phase cell cycle arrest.

### The dichloromethane extract of *F. crocata* increased the p53, procaspase-8 and procaspase-3 expression

**Fig. 7:**
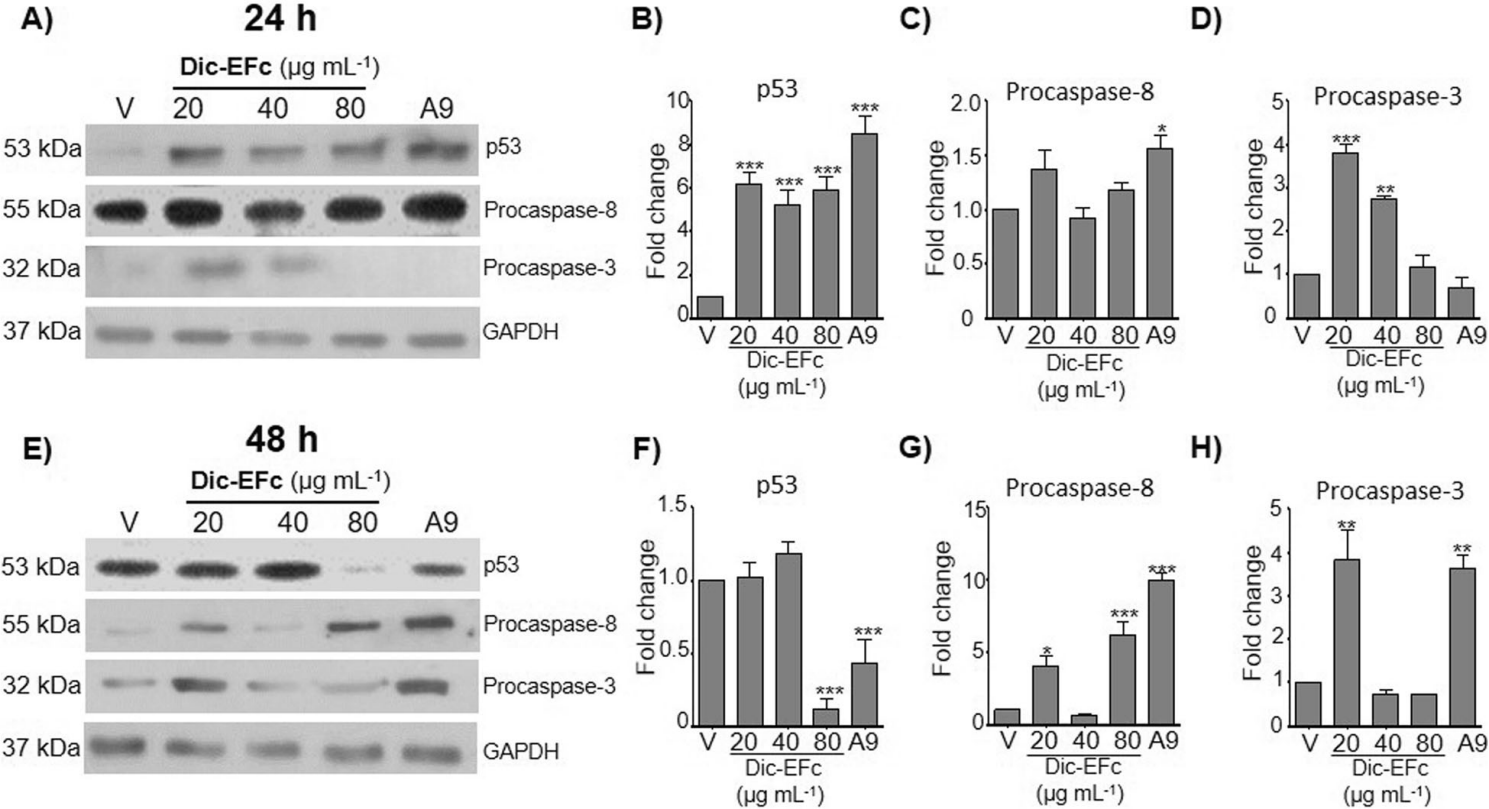
Dic-EFc and the A9-Dic-EFc fraction increased p53, procaspase-8 and procaspase-3 expression. Top panel: 24 h of treatment; Bottom panel: 48 h of treatment. Representative western blot of p53, procaspase-8, procaspase-3 and GAPDH, after 24 h (**a**) and 48 h (**e**) of treatment with Dic-EFc or A9-Dic-EFc fraction. (**b**, **c** and **d**) Densitometric analysis of (**a**). V, vehicle: cells treated with diluent of extract, 1% DMSO. (**f**, **g** and **h**) Densitometric analysis of (**e**). One-way ANOVA, Dunnett’s test: *p < 0.05; **p < 0.01 and ***p < 0.001 versus V

MDA-MB-231 cells were treated with 0–80 μg mL^− 1^ of Dic-EFc and 80 μg mL^− 1^ of the A9-Dic-EFc fraction to determine changes in expression of proteins such as p53, procaspase-8, and procaspases-3, which are associated with apoptosis and the cell cycle (Fig. 7). It was observed that the Dic-EFc and A9 fraction increased p53 expression at 24 h of treatment (Fig. 7b), although it markedly decreased p53 expression at 48 h (Fig. 7f). The increase in p53 expression could be associated with the diminution of cell number in the G1 and S phases of the cell cycle. With respect to caspase expression, in Western blot assays, we observed bands around 32 kDa and 55 kDa, which correspond to procaspase-3 and procaspase-8, respectively (Fig. 7a and e). Dic-EFc increased procaspase-8 expression at 48 h of treatment only at the 80 μg mL^− 1^ concentration (Fig. 7g). Minor concentrations (20–40 μg mL^− 1^) of Dic-EFc increased procaspase-3 expression at 24 h and 48 h of treatment (Fig. 7d and h). On the other hand, the A9-Dic-EFc fraction increased procaspase-8 expression at 24 h and 48 h of treatment (Fig. 7c and g), and procaspase-3 expression at 48 h (Fig. 7h). It is possible that accumulated procaspase-3 and procaspase-8 are being processed into active forms—i.e., caspase 8 and caspase 3, respectively— which contribute to the progression of apoptosis induced mainly by treatment with the A9-Dic-EFc fraction in MDA-MB-231 cells.

## Discussion

TNBC represents 15–20% of breast cancer cases and is related to poor prognosis, metastasis, and death. This type of breast tumor does not respond to conventional chemotherapy because the cells do not express ER and PR and lack overexpression of the HER2 protein, thus representing the sub-type with worst prognosis among breast cancer cases [66]. Current research is focused on the chemical compounds of plants and their effect on cellular models of cancer [14]. It has been observed that natural products obtained from plants exhibit antiproliferative, antimigratory, and anti-invasive have effects on cancer cells and could be used as adjuvant therapies in cancer [12–15]. Studies conducted in species of the *Ficus* genus have revealed the greatest antiproliferative potential in cervical, hepatic, leukemia, lung, and colon-cancer cell lines [43, 45, 47, 50]. It has been described that this activity is due to the presence of compounds such as alkaloids, flavonoids, coumarins, phenols, steroids, terpenoids, and triterpenoids [16, 18, 21, 23–26, 39, 40, 51, 52, 54, 56, 58, 59]. In Mexico, *Ficus* species are distributed throughout the country [60, 61, 67], yet there have been no studies, to our knowledge, on the chemical composition or biological properties of the *Ficus* species distributed in Mexico. In this study, we analyzed the phytochemical profile of leaf extracts of *F. crocata* and their effect on the cell proliferation of TNBC MDA-MB-231 cells.

This is the first study, to our knowledge, to evaluate the cytotoxic activity of leaf extracts of *F. crocata*. We observed that Hex-EFc, Dic-EFc, and Ace-EFc decreased the proliferation of MDA-MB-231 cells, with Ace-EFc and mainly Dic-EFc being the most active. An interesting observation in this study was that Dic-EFc did not show a cytotoxic effect on MCF-10A non-tumor cells at the same concentrations that affected the viability of MDA-MB-231 cells (5–80 μg mL^− 1^), which supports the possible use of extracts of *F. crocata* as an alternative therapy against breast tumors. However, this observation must be analyzed further. These observations are in agreement with other studies, which reported a decrease of cell viability in the breast cancer cell lines T47D and MDA-MB-231 exposed to the leaf extracts of *Ficus septica* Burm. and *Ficus carica* [56, 68].

To separate compounds into groups based on their polarity, Dic-EFc was fractionated and we observed that the A9-Dic-EFc, A12-Dic-EFc, and A13-Dic-EFc fractions exerted a greater effect in terms of decreasing the viability of MDA-MB-231 cells. A9-Dic-EFc was the most effective of the fractions, as compared to Dic-EFc. This effect suggests that isolation of compounds increased their biological activity. The phytochemical composition of Dic-EFc and the A9-Dic-EFc, A12-Dic-EFc, and A13-Dic-EFc fractions revealed the presence of alkaloids, coumarins, lignans, anthraquinones, phenols, terpenoids, and triterpenoids. The presence of these compounds has already been reported for the *Ficus* genus, and it has been demonstrated that these compounds possess biological activity [16, 46, 56, 59].

We observed that the exposure of MDA-MB-231 cells to *F. crocata* extracts induced morphological changes such as a decrease in cell size, a rounded shape, and the formation of intracellular vacuoles. In a concentration-dependent manner, Dic-EFc and A9-Dic-EFc decreased the cell population in the S and G2/M phases of the cell cycle and increased the cell population in the sub-G0, which was consistent with the increase of apoptotic cells that could explain the morphologic changes in the cells and the decrease in cell viability. The apoptotic effect of Dic-EFc was potentiated with the A9-Dic-EFc, A12-Dic-EFc, and A13-Dic-EFc fractions, inducing apoptosis in around 50% of the cell population. In this regard, we observed that Dic-EFc and the A9-Dic-EFc fraction increased p53 expression at 24 h of treatment, which could be associated with apoptosis and cell cycle arrest, but curiously, p53 expression markedly decreased after of 48 h with 80 μg mL^− 1^ of Dic-EFc and A9-Dic-EFc. This increase and a subsequent decrease in the expression of p53 has also been observed in another study with HCT116 cells treated with betulinic acid (BA), in which the treatment also induced apoptosis, suggesting that other proteins could be involved in BA-induced apoptosis in addition to p53 [69]. Moreover, in breast cancer cell lines MCF-7 and MDA-MB-231, it was observed that *Vernonia amygdalina* (VA) extracts induced apoptosis and cell cycle arrest in a p53-independent manner. Interestingly, these authors observed that VA increased p53 expression in a time-dependent manner in MCF-7 cells, whereas in MDA-MB-231, p53 expression decreased after 48 h [70]. We observed a similar event in our study. On the other hand, the A9-Dic-EFc fraction increased the expression of procaspase-8 and procaspase-3, which are initiator and executioner caspases, respectively, involved in the extrinsic apoptosis pathway [71]. These proteins could be processed in their active form and induce apoptosis, which would explain the increase in the apoptotic cell population induced mainly by the A9-Dic-EFc fraction in MDA-MB-231 cells. Dic-EFc also increased procaspase-8 expression at 48 h of treatment at an 80 μg mL^− 1^ concentration, while 20–40 μg mL^− 1^ concentrations increased procaspase-3 expression, which could be associated with the slight increase in the apoptotic cell population under these treatment conditions. It is probable that the apoptosis induced by the extracts of *F. crocata* and its fractions in MDA-MB-231 cells is through an p53-independent pathway, such as PUMA protein for example, which is known to be a powerful apoptosis inducer associated or not with p53 [72]. However, this hypothesis should still be tested.

Our observations are consistent with those of other studies. Zhang et al. (2018) reported that *F. carica* extracts induce apoptosis and cell cycle arrest at the S phase in MDA-MB-231 cells, increasing the expression of genes that promote apoptosis and the regulation of the cell cycle, such as *BAX*, *TP53*, and *TP21* [68]. Similarly, in a study by Choudhari et al., the authors reported that the aqueous extract of *Ficus religiosa* induces cell cycle arrest in the G1 phase in SiHa cells, accompanied by an increase in the expression of p53, p21, and pRb proteins. Moreover, in HeLa cells, the extract induces apoptosis through an increase in intracellular Ca^2+^, leading to the loss of mitochondrial membrane potential, the release of cytochrome-C, and an increase in caspase-3 expression [47]. It has also been reported that the flavonoid quercetin induces apoptosis in HeLa cells, promoting the accumulation of reactive oxygen species (ROS), upregulating proapoptotic proteins such as cytochrome C, Apaf-1, and caspases, and downregulating the antiapoptotic proteins Bcl-2 and survivin [73, 74].

The primary compound in Dic-EFc was lupeol, a triterpene also present in Ace-EFc and Hex-EFc, although in smaller proportions (Table 1). Triterpenes were only present in the A9-Dic-EFc fraction, which suggests that lupeol was only present in A9-Dic-EFc and not in the A12- and A13-Dic-EFc fractions. It has been reported that lupeol induces apoptosis and cell cycle arrest in several cancer cell lines. For example, in pancreatic cancer cells, lupeol induces apoptosis by decreasing the levels of p-AKT and p-ERK, as well as cell cycle arrest in the G0/G1 phase, by upregulating P21 and P27 and downregulating cyclin D1 proteins [75]. A similar effect was observed in osteosarcoma cells MNNG/HOS and MG-63 [76]. In gallbladder carcinoma GBC-SD cells, lupeol was shown to induce apoptosis and inhibit invasion by downregulating the activity of p-EGFR and MMP-9 [77]. In non-small cell lung cancer cells, it was observed that lupeol inhibits the phosphorylation of EGFR by binding to its tyrosine kinase domain and reducing STAT3 phosphorylation, which contributes to the induction of apoptosis [78]. In the hepatocarcinoma cell lines SMMC7721 and HepG2 as well as lung carcinoma A427 cells, lupeol was shown to induce low expression of the antiapoptotic protein Bcl-2 and upregulate the proapoptotic protein BAX [79, 80]. Considering the previously noted observations, it is possible that the metabolites present in *F. crocata* extracts, such as lupeol, probably in synergy with other metabolites, induce cell cycle arrest and apoptosis in MDA-MB-231 cells, altering the expression of cell cycle regulatory proteins such as p53, p21, p27, and cyclins, as well as the expression of anti- and proapoptotic proteins, such as caspases, PUMA, Bax, and Bak. However, this hypothesis still needs to be further analyzed.

One limitation of this study is that the assays were executed in only one breast cancer cell line. It would be important to evaluate the effect of *F. crocata* extracts on a larger number of tumor and non-tumor cell lines. Furthermore, the effect of pure metabolites isolated from *F. crocata* extracts, such as lupeol, Stigmastan, and others, has not, to our knowledge, been tested. This effect will be evaluated in a subsequent study. Our observations comprise, to our knowledge, a first approach to investigating the possible use of extracts of *F. crocata* as alternative or complementary therapy against cancer. Nonetheless, their cytotoxicity and molecular mechanisms must be analyzed in more study models.

## Conclusions

The leaf extract of *F. crocata* decreases the proliferation capacity of MDA-MB-231 triple-negative breast cancer cells, decreasing the S and G2/M cell cycle phases, and inducing apoptosis possibly through an p53-independent pathway. By fractionating the extract, the antiproliferative activity against MDA-MB-231 cells can be further potentiated. These findings provide information on the biological activity of *F. crocata* extracts and suggest their potential use as an alternative or complementary therapy against triple-negative breast cancer. Nevertheless, more studies are required to determine the components responsible for this biological activity, as well as the molecular and cellular mechanisms involved.

## Supplementary information

**Additional file 1: Fig. S1.** Structures of the molecules identified by GC/MS from the Hex-EFc. The numbers correspond to name of compounds shown in Table 1.

**Additional file 2: Fig. S2.** Structures of the molecules identified by GC/MS from the Dic-EFc. The numbers correspond to name of compounds shown in Table 1.

**Additional file 3: Fig. S3.** Structures of the molecules identified by GC/MS from the Ace-EFc. The numbers correspond to name of compounds shown in Table 1.

**Additional file 4: Fig. S4.** Half-maximal inhibitory concentration (IC_50_) of leaf *F. crocata* extracts and fractions on cell proliferation (MTT assay).

**Additional file 5: Fig. S5.** Effect of Dic-EFc on MCF-10A cell viability after 48 h of treatment. MTT assay; V: vehicle, DMSO. (−): negative control, 5% FBS. (+): positive control, 100 μM Ara-C (cytarabine); Dic-EFc: dichloromethane extract of *F. crocata*. One-way ANOVA, Dunnett’s test: **p < 0.05, **p < 0.01 and ***p < 0.001 versus V.

**Additional file 6: Table S1.** Phytochemical profile of the dichloromethane extract fractions of *F. crocata* leaves.

**Additional file 7: Fig. S6.** Full Images of the blots shown in Fig. 7. V: Vehicle (DMSO), B: Basal, cells without treatment. Arrow: row of bands corresponding to p53, procaspase-8, procaspase 3 and GAPDH shown in Fig. 7. Red box: data not shown in Fig. 7; The Basal condition was omitted.

## Data Availability

All data generated or analyzed during this study are included in this published article and its supplementary information files.

## Abbreviations

Hex-EFc: Hexane extract of *F. crocata*
Dic-EFc: Dichloromethane extract of *F. crocata*
Ace-EFc: Acetone extract of *F. crocata*
GC-MS: Gas chromatography coupled with mass analysis
TNBC: Triple-negative breast cancer
ER: Estrogen receptors
PR: Progesterone receptors
OCC: Open glass Column Chromatography
DMEM: Dulbecco’s Modified Eagle Medium
FBS: Fetal bovine serum
DMSO: Dimethyl Sulfoxide
Ara-C: Cytarabine, cytosine β-D-arabinofuranoside
PBS: Phosphate-Buffered Saline
RIPA: Radioimmunoprecipitation assay buffer
HEPES: 4-(2-hydroxyethyl)-1-piperazineethanesulfonic acid
EDTA: Ethylene-diamine-tetraacetic acid
EGTA: Ethylene glycol tetraacetic acid
OD: Optical density
IC_50_: Half-maximal inhibitory concentration
PI: Propidium iodide
RT: Room temperature
SD: Standard deviation
ANOVA: One-way analysis of variance
