# Supplementary figures and images for: Phytochemical profile and antiproliferative effect of Ficus crocata extracts on triple-negative breast cancer cells
Source: BMC Complement Med Ther. 2020 Jun 22;20:191. doi: 10.1186/s12906-020-02993-6 (PMC7309984; doi:10.1186/s12906-020-02993-6)

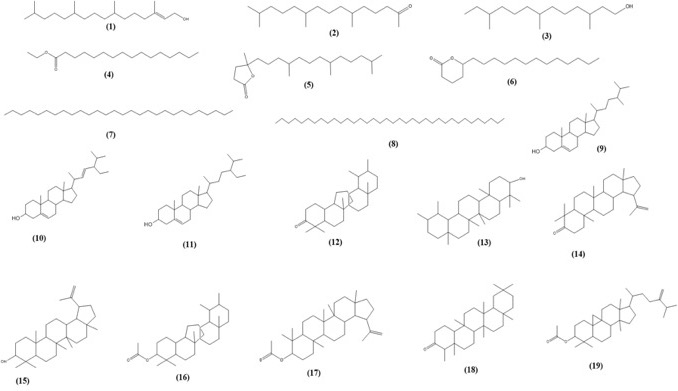

Supplement: Supplementary file 1 — Additional file 1: Fig. S1. Structures of the molecules identified by GC/MS from the Hex-EFc. The numbers correspond to name of compounds shown in Table 1. [file 12906_2020_2993_MOESM1_ESM.jpg]

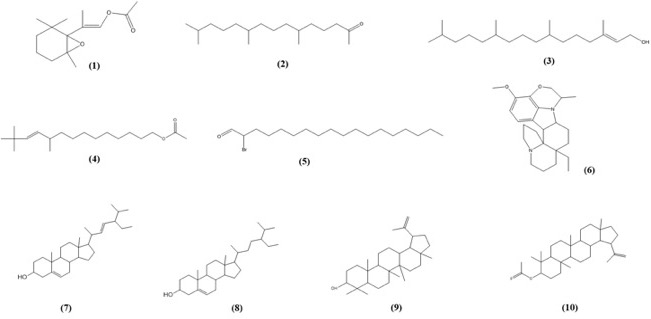

Supplement: Supplementary file 2 — Additional file 2: Fig. S2. Structures of the molecules identified by GC/MS from the Dic-EFc. The numbers correspond to name of compounds shown in Table 1. [file 12906_2020_2993_MOESM2_ESM.jpg]

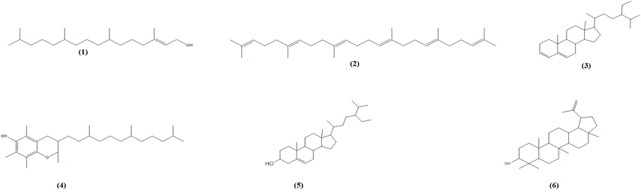

Supplement: Supplementary file 3 — Additional file 3: Fig. S3. Structures of the molecules identified by GC/MS from the Ace-EFc. The numbers correspond to name of compounds shown in Table 1. [file 12906_2020_2993_MOESM3_ESM.jpg]

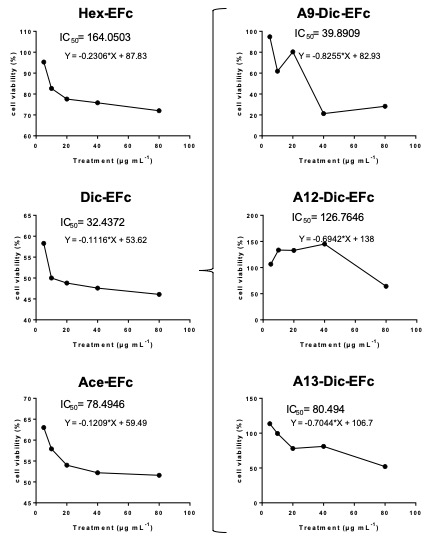

Supplement: Supplementary file 4 — Additional file 4: Fig. S4. Half-maximal inhibitory concentration (IC50) of leaf F. crocata extracts and fractions on cell proliferation (MTT assay). [file 12906_2020_2993_MOESM4_ESM.jpg]

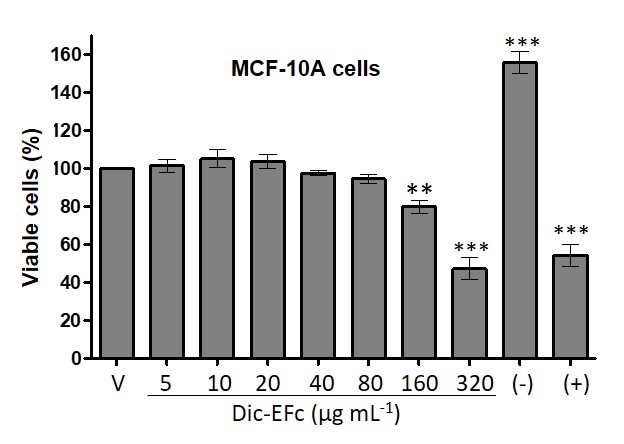

Supplement: Supplementary file 5 — Additional file 5: Fig. S5. Effect of Dic-EFc on MCF-10A cell viability after 48 h of treatment. MTT assay; V: vehicle, DMSO. (−): negative control, 5% FBS. (+): positive control, 100 μM Ara-C (cytarabine); Dic-EFc: dichloromethane extract of F. crocata. One-way ANOVA, Dunnett’s test: **p < 0.05, **p < 0.01 and ***p < 0.001 versus V. [file 12906_2020_2993_MOESM5_ESM.jpg]

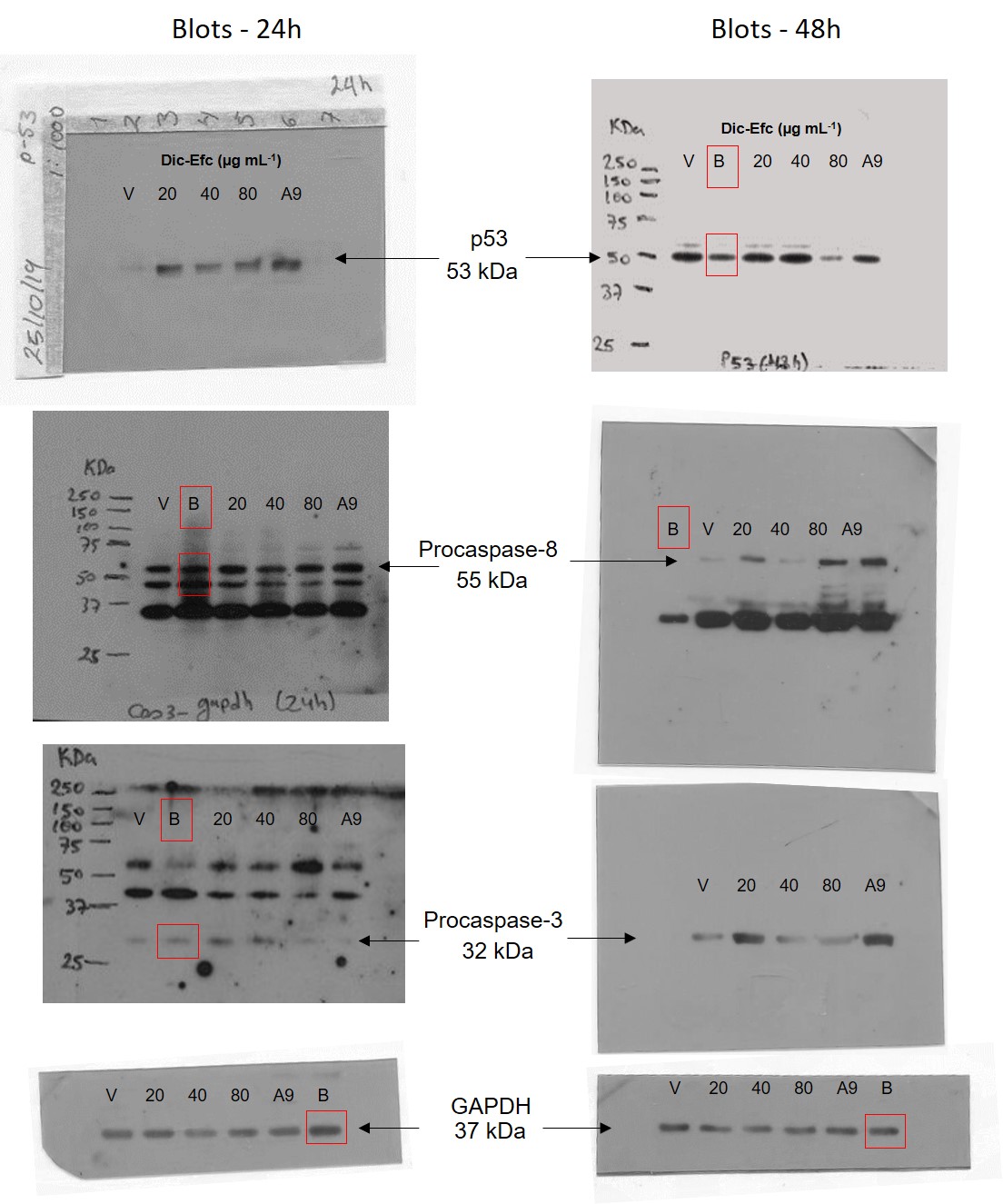

Supplement: Supplementary file 7 — Additional file 7: Fig. S6. Full Images of the blots shown in Fig. 7. V: Vehicle (DMSO), B: Basal, cells without treatment. Arrow: row of bands corresponding to p53, procaspase-8, procaspase 3 and GAPDH shown in Fig. 7. Red box: data not shown in Fig. 7; The Basal condition was omitted. [file 12906_2020_2993_MOESM7_ESM.jpg]
