# Supplementary material for: Phytochemical profile and antiproliferative effect of Ficus crocata extracts on triple-negative breast cancer cells
Source: BMC Complement Med Ther. 2020 Jun 22;20:191. doi: 10.1186/s12906-020-02993-6 (PMC7309984; doi:10.1186/s12906-020-02993-6)
Supplement: Supplementary file 6 — Additional file 6: Table S1. Phytochemical profile of the dichloromethane extract fractions of F. crocata leaves. [file 12906_2020_2993_MOESM6_ESM.pdf]

Table S1: Phytochemical profile of the dichloromethane extract fractions of *F. crocata* leaves.

Fractions	Alkaloids	Anthraquinones	Anthrones	Coumarins	Essential oils	Phenylpropanoids	Terpenoids	Lignans	Cardiac glycosides	Curcubitacins	Triterpenes	Steroids	Saponins
A1	-	+	-	+	-	-	+	+	+	+	+	+	-
A2	+	+	-	+	-	-	-	+	+	-	+	+	+
A3	+	+	-	+	+	+	-	+	+	-	+	+	+
A4	+	+	-	+	+	+	-	+	+	-	+	+	+
A5	+	+	-	-	+	+	+	+	+	+	+	+	+
A6	+	-	+	+	+	+	+	+	+	+	+	+	+
A7	+	+	-	-	-	-	+	+	+	+	+	+	+
A8	-	+	-	-	-	-	+	+	+	+	+	+	+
A9	-	+	-	-	+	+	+	+	+	+	+	+	+
A10	-	+	-	-	+	+	+	+	+	+	+	+	+
A11	-	+	-	-	+	+	+	-	-	+	-	-	-
A12	-	+	-	-	-	-	-	-	-	-	-	-	-
A13	-	+	-	-	-	-	-	+	+	-	-	-	-
A14	+	+	-	-	-	-	-	+	+	-	-	-	-
A15	+	+	-	-	-	-	-	+	+	-	-	-	-
A16	+	+	-	-	-	-	-	+	+	-	-	-	-
A17	+	+	-	-	-	-	-	+	+	-	-	-	-
